# Fabrication of Low-Cost Porous Carbon Polypropylene Composite Sheets with High Photothermal Conversion Performance for Solar Steam Generation

**DOI:** 10.3390/polym16192813

**Published:** 2024-10-04

**Authors:** Shuqing Xu, Shiyun Wu, Bin Xu, Jiang Ma, Jianjun Du, Jianguo Lei

**Affiliations:** 1 College of Mechatronics and Control Engineering, Shenzhen University, Shenzhen 518061, China; 2 School of Mechanical Engineering and Automation, Harbin Institute of Technology Shenzhen, Shenzhen 518055, China

**Keywords:** ultrasonic pressing, photothermal conversion, porous structure, solar steam generation, desalination

## Abstract

The development of absorber materials with strong light absorption properties and low-cost fabrication processes is highly significant for the application of photothermal conversion technology. In this work, a mixed powder consisting of NaCl, polypropylene (PP), and scale-like carbon flakes was ultrasonically pressed into sheets, and the NaCl was then removed by salt dissolution to obtain porous carbon polypropylene composite sheets (P-CPCS). This process is simple, green, and suitable for the low-cost, large-area fabrication of P-CPCS. P-CPCS has a well-distributed porous structure containing internal and external connected water paths. Under the dual effects of the carbon flakes and porous structure, P-CPCS shows excellent photothermal conversion performance in a broad wavelength range. P-CPCS-40 achieves a high temperature of 128 °C and a rapid heating rate of 12.4 °C/s under laser irradiation (808 nm wavelength, 1.2 W/cm^2^ power). When utilized for solar steam generation under 1 sun irradiation, P-CPCS-40 achieves 98.2% evaporation efficiency and a 1.81 kg m^−2^ h^−1^ evaporation rate. This performance means that P-CPCS-40 outperforms most other previously reported absorbers in terms of evaporation efficiency. The combination of carbon flakes, which provide a photothermal effect, and a porous polymer structure, which provides light-capturing properties, opens up a new strategy for desalination, sewage treatment, and other related fields.

## 1. Introduction

The shortage of water resources is a major challenge for humanity [1]. Globally, clean drinking water is still not accessible for over 700 million people [2]. Therefore, the investigation of more efficient strategies for obtaining clean drinking water is a global research topic [3,4,5]. Numerous scholars have reported that solar-powered water evaporation is an important tool for ameliorating water scarcity and water pollution [6,7,8]. Solar water evaporators are systems containing an absorber material that can strongly absorb light. The choice of absorber material and structure directly affects the efficiency of water evaporation. So far, numerous materials have been reported to exhibit high evaporation efficiencies [9], including plasma metal nanoparticles [10,11,12,13], semiconductors [14,15], and carbon [16,17,18,19]. Among plasma metal nanoparticles, gold nanoparticles are the most widely studied due to their excellent photothermal conversion efficiency and structural controllability [20,21]. However, the high cost of gold has limited the widespread application of gold nanoparticles. Semiconductors have the advantages of high stability and good photothermal conversion efficiency. Among the known semiconductor materials, metal oxides and sulfur compounds such as Ti_2_O_3_, black TiO_2_, and MoS_2_ have been widely used in solar water evaporation [22,23]. However, the preparation process of semiconductor absorbers is complex and cumbersome [24,25,26]. Carbon materials have the advantages of abundance, stability, environmental friendliness, and superior photothermal conversion efficiency [27]. Consequently, various structural forms of carbon materials have been widely studied and applied in solar water evaporation, including porous graphene sheets [28], carbon nanoparticles [29], carbon nanotubes [30], 3D graphene nanofluids [31], and graded graphene foams [32]. However, pure carbon absorbers are poorly hydrophilic and lack sufficient flexibility, resulting in inefficient water evaporation and difficulties in large-area fabrication.

Polymers have benefits such as corrosion resistance, low thermal conductivity, biocompatibility, and easy processing [33]. Therefore, strategies for polymerization or combining polymers with photothermal materials can achieve enhanced corrosion resistance, photothermal conversion, and water evaporation properties [34,35]. Huang et al. [36] reported solar evaporators with distinctive dendritic micro- and nanostructures in which Au nanoparticle absorbers were attached to a melamine sponge skeleton coated with silicon nanoparticles. Wang et al. [37] prepared a three-dimensional MoS_2_-based aerogel that achieved high evaporation efficiency by cross-linking chitosan and MoS_2_ nanosheets polymerized with polydopamine via a liquid-phase one-pot method. Finnerty et al. [38] prepared a nature-inspired synthetic graphene oxide (GO) leaf by cross-linking triethylenetetramine (TETA) and 1,4-butanediol diglycidyl ether (BDGE) with GO nanosheets. This cross-linked material was loaded on polystyrene sheets to form a solar water evaporator. Cui et al. [39] designed a porous graphene sponge structure with an incorporated graphene film (PGS/GF) obtained by cryostructuring and laser processing techniques. This structure was utilized as an absorber, and polystyrene foam was utilized as a support and insulation layer. These materials and other reported composite absorbers prepared by combining photothermal materials and polymers show excellent photothermal conversion performance and water evaporation efficiency. However, their complex structures and cumbersome preparation processes have severely limited their large-scale fabrication and application. Therefore, new absorber materials still need to be developed to meet the demand for large-scale, low-cost, and highly efficient photothermal applications.

In this paper, porous carbon polypropylene composite sheets (P-CPCS) were prepared by ultrasonic pressing and salt dissolution for efficient solar steam generation. The presence of scale-like carbon flakes and porous structures provides excellent photothermal conversion performance and water evaporation efficiency. Moreover, the low density of polypropylene (PP) is conducive to the self-floating of P-CPCS in water bodies. In addition, the precursor materials used to prepare P-CPCS are low cost and accessible, and the P-CPCS preparation process is simple, pollution-free, and suitable for large-area production.

## 2. Materials and Methods

### 2.1. Materials and Equipment

Polypropylene powder (~150 μm average particle size, Korea Chungnam Lotte Chemical Co.) was selected as the substrate material. Scale-like carbon flakes (thickness of ~200 nm, average diameter of ~5 μm, Shenzhen Hanhui Graphite Co., Shenzhen, China) were selected as the photothermal material. The flake morphology is shown in Figure S1. NaCl powder (~7.5 μm average particle size) was obtained by recrystallization to prepare porous structures. In brief, a saturated aqueous NaCl solution was added to an ethanol solution under vigorous stirring to achieve complete mixing and recrystallization. The recrystallized NaCl particles were collected and dried under vacuum, then ground and sieved to obtain NaCl powder. The particle morphology and particle size distributions are shown in Figure S2a,b, respectively.

An ultrasonic loading system was utilized in this study. This system contained a pneumatic piston, horn, transducer, worktable, air source, and power supply. The resonant frequency and maximum amplitude of ultrasonic vibration were 20 kHz and 60 μm, respectively.

### 2.2. Fabrication of P-CPCS

**Figure 1:**
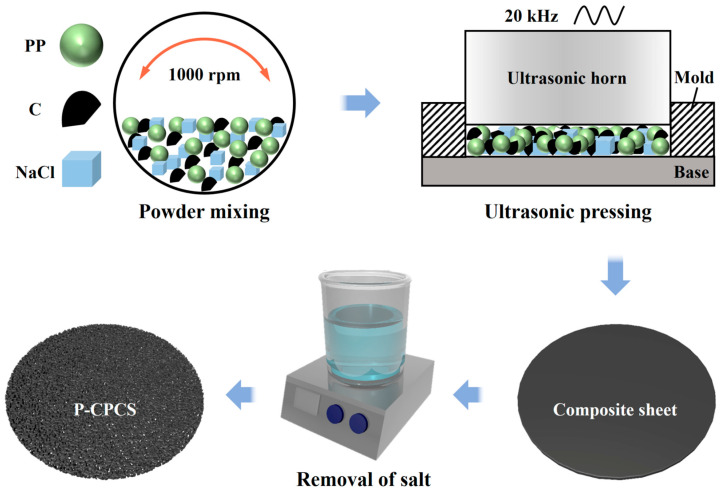
Schematic illustration for the preparation of P-CPCS.

As shown in Figure 1, the preparation process of P-CPCS consisted of three main stages. (1) Powder mixing. PP powder, carbon flakes, and NaCl powder were mixed in different proportions. (2) Ultrasonic pressing (UP). A mold cavity was filled with the mixed powder, and ultrasonic pressing was initiated. The high-frequency vibrations of the ultrasonic horn provided viscoelastic and friction heat, which caused the PP powder to rapidly plasticize and melt [40,41,42]. At the same time, the carbon flakes and salt powder were bonded together by the PP melt to form a sheet under the pressure of the ultrasonic horn. (3) Removal of salt. Each formed composite sheet was soaked in water for the removal of NaCl.

In this study, the volume ratio of carbon flakes was fixed at 10%. The NaCl powder content directly affected the formability and properties of the P-CPCS. Therefore, 30%, 40%, and 50% volume ratios of NaCl powder were employed to study the formability and properties of the corresponding P-CPCS samples. The samples prepared using different NaCl powder proportions were denoted CPCS (0% NaCl), P-CPCS-30 (30% NaCl), P-CPCS-40 (40% NaCl), and P-CPCS-50 (50% NaCl). The ultrasonic loading system was utilized with a default resonant frequency of 20 kHz and the following parameters: 500 N ultrasonic trigger force, 300 kPa loading pressure, 60 μm amplitude, and 5 s pressure holding time. The duration time of ultrasonication was adjusted according to the forming effect of P-CPCS. Before ultrasonic pressing, PP powder, carbon flakes, and NaCl powder were mixed in a planetary mixer at 1000 rpm for 1 h. After ultrasonic pressing, the formed composite sheets were soaked in water at 80 °C for 4 h to remove NaCl powder.

### 2.3. Characterization

The morphologies of the prepared porous structures were studied using scanning electron microscopy (SEM, Quanta FEG450, FEI, Hillsboro, OR, USA). Each sample was coated with gold prior to SEM analysis. Sample porosities were evaluated using a pore size distribution meter (Autopore V 9620, Micromeritics, Norcross, GA, USA). X-ray diffractometry (XRD, MiniFlex600, Rigaku, Akishima-shi, Japan) was performed in the range of 10–90° to confirm the complete removal of NaCl from P-CPCS after soaking. Fourier transform infrared (FTIR) spectra were obtained in the wavelength range of 400 to 4000 cm^−1^ on an FTIR spectrometer (Nicolet 6700, Nicolet, Madison, WI, USA). Reflection spectra were obtained in the range of 380 to 900 nm on a microspectroscopy system (Lambda950, PerkinElmer, Waltham, MA, USA) under integrating sphere mode. The water contact angles (WCAs) of the samples were determined using 2.5 μL water droplets and a drop shape analysis instrument (DSA100S, Krüss, Hamburg, Germany).

The photothermal conversion performance of the P-CPCS and CPCS samples was evaluated by vertically irradiating each sample on a quartz stage with a laser. An infrared (IR) imaging camera (Fotric 280d) was utilized to monitor variations in surface temperature with a data acquisition frequency of 1 Hz. Laser irradiation was performed at wavelengths of 1064 nm (PGL-V-H-1064), 808 nm (5 W, fiber-tailed, multimode diode laser), 655 nm (PGL-V-H-655), 532 nm (PGL-V-H-532), and 405 nm (PGL-V-H-405). Each laser irradiation cycle lasted 100 s, and the effective irradiation time per cycle was 50 s. Irradiation was performed for a total of 4.5 cycles. To study the high-temperature stability of the P-CPCS photothermal conversion performance, irradiation was performed using the 808 nm laser at different power settings (0.4, 0.6, 0.8, 1, 1.2 W/cm^2^).

The water evaporation performance of P-CPCS was characterized using a Xe light system (CEL-HXF 300, Beijing Education Au-light Co., Ltd., Beijing, China). To simulate sunlight, this system was placed on top of each tested sample, and an AM 1.5 filter was used to filter the light. The temperature of each sample in air and water was recorded using the Fotric 280d IR camera. A computer-operated electronic balance with an accuracy of measurement accuracy of 0.01 mg (Quintix35–1CN, Sartorius, Göttingen, Germany) was utilized to track the mass loss of water due to evaporation. Light intensity was monitored using an optical power meter (CEL-NP2000, Beijing Education Au-light Co., Ltd., Beijing, China).

Ion (Na^+^, K^+^, Ca^2+^, Mg^2+^) concentrations were measured using an inductively coupled plasma emission spectrometer (Agilent 720ES, Agilent, Santa Clara, USA). The absorbance spectra of the methylene blue (MB) solution and the water collected by evaporation after irradiation of the MB solution were obtained using a UV–Vis–NIR spectrophotometer (Shimadzu UV-1280, Shimadzu, Kyoto, Japan) in the range of 380 to 800 nm.

## 3. Results and Discussion

### 3.1. Formability and Characterization of P-CPCS

**Figure 2:**
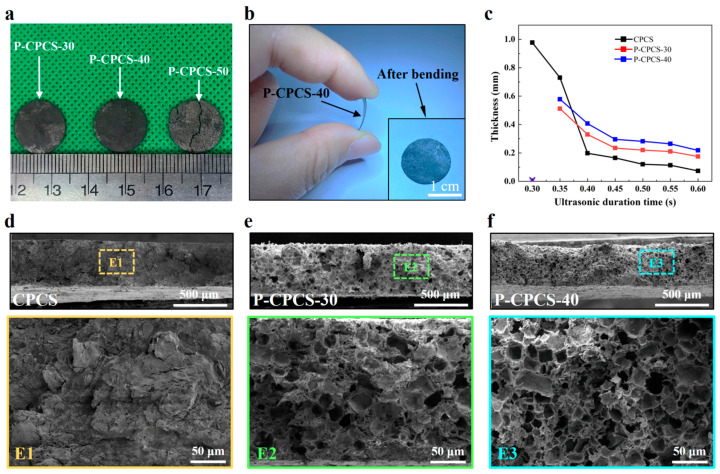
Formability and structural characteristics of P-CPCS. (**a**) Photographs of P-CPCS-30, P-CPCS-40, and P-CPCS-50. (**b**) P-CPCS-40 in bending state and after bending multiple times. (**c**) Variation in thickness of P-CPCS with ultrasonic duration time. Cross-section SEM images and magnified views of (**d**) CPCS, (**e**) P-CPCS-30, and (**f**) P-CPCS-40.

Higher NaCl content is beneficial for increasing the porosity of the prepared composite sheets. However, the NaCl content also affects the formability and strength of these composite sheets. The formability of the prepared sheets was studied by utilizing three powder mixtures (NaCl volume content of 30%, 40%, and 50%) for ultrasonic pressing. The formed P-CPCS-30, P-CPCS-40, and P-CPCS-50 composite sheets obtained after dissolving the NaCl powder are shown in Figure 2a. P-CPCS-50 shows clear and significant cracks, indicating incomplete and defective composite sheet formation under a NaCl content of 50%. As displayed in Figure 2b, P-CPCS-40 is still intact after multiple bending cycles, which indicates that this sample has sufficient strength and good forming quality.

P-CPCS-30 and P-CPCS-40 are unable to be pressed into sheets with an ultrasonic duration time of just 0.3 s, as shown in Figure 2c. The thicknesses of CPCS, P-CPCS-30, and P-CPCS-40 become smaller as the ultrasonic duration time increases. In addition, for mixed powders with the same total weight and different NaCl content, the thickness of the prepared composite sheets is different for the same ultrasonic duration time. Higher NaCl content leads to a larger thickness when the ultrasonic duration time is greater than 0.4 s. As the ultrasonic duration time increases, more of the mixed powder melt is extruded through the gap between the ultrasonic horn and the mold, leading to a reduced thickness. However, for the samples with higher NaCl content, the melt is less fluid, meaning that less of the melt is extruded and the obtained sheets have larger thicknesses. To provide a better performance comparison, different ultrasonic duration times were used to prepare CPCS (0.39 s), P-CPCS-30 (0.42 s), and P-CPCS-40 (0.45 s) samples with equal thicknesses of about 300 μm.

**Figure 3:**
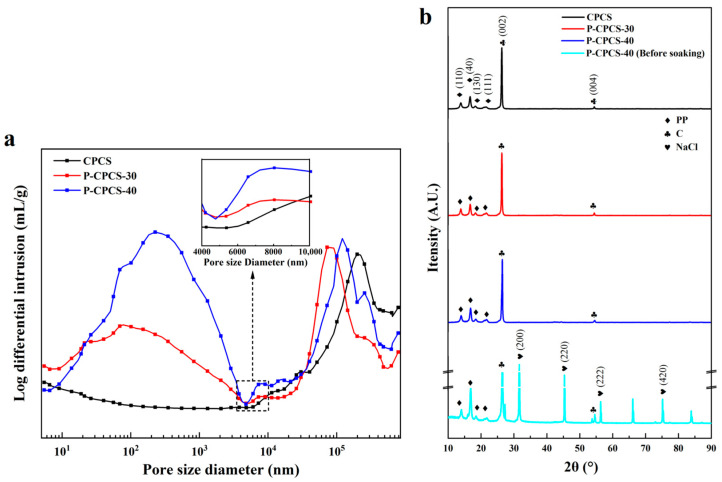
(**a**) Pore size distributions of CPCS, P-CPCS-30, and P-CPCS-40 and (**b**) XRD patterns.

**Figure 4:**
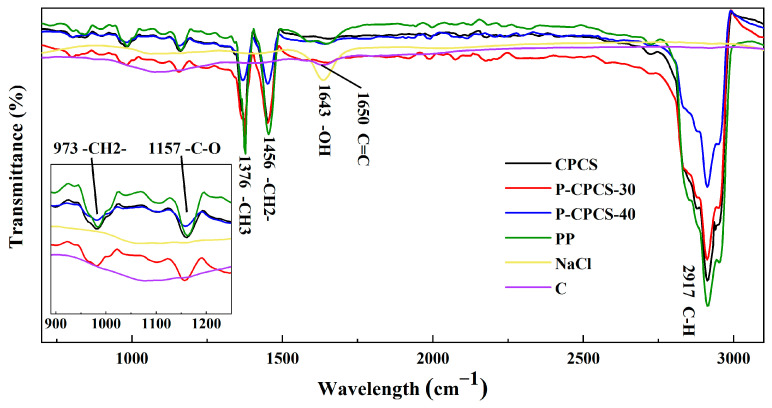
FTIR spectra of raw materials, CPCS, P-CPCS-30, and P-CPCS-40.

The cross-sectional morphologies of CPCS, P-CPCS-30, and P-CPCS-40 are displayed in Figure 2d–f. CPCS does not show the presence of any significant pores, while many pores can be observed in P-CPCS-30 and P-CPCS-40. Moreover, P-CPCS-40 has more pores than P-CPCS-30. These pores are uniformly distributed in the P-CPCS-30 and P-CPCS-40 composite sheets, providing a good basis for water path connectivity. The porosities of CPCS, P-CPCS-30, and P-CPCS-40 were respectively determined to be 22.09%, 36.52%, and 46.39% according to mercury intrusion. The pore distributions shown in Figure 3a indicate that the difference in porosity among CPCS, P-CPCS-30, and P-CPCS-40 is mainly in the pore size range of 0.01–10 μm. Within this pore size range, P-CPCS-40 has significantly more pores than P-CPCS-30, while P-CPCS-30 has significantly more pores than CPCS. Apparently, there are micron pores and nanopores in both P-CPCS-30 and P-CPCS-40. The micron pores were mainly formed by the dissolution of micron-scale NaCl particles and agglomerates of multiple NaCl particles in water. The nanopores were formed in three ways. In the first, nanopores are formed by the dissolution of small amounts of nanoscale NaCl particles. In the second, nanopores are produced when the PP melt is unable to completely cover the surface of the NaCl particles or the carbon flakes. In the last, nanopores are left behind when the PP melt cannot completely fill the gaps created by the mutual support of the NaCl particles and the carbon flakes. The latter two ways are the main reasons for the formation of a large number of nanopores in P-CPCS-30 and P-CPCS-40. The XRD pattern of P-CPCS-40 before soaking clearly shows the characteristic peaks of NaCl at 31.77°, 45.54°, 56.60°, and 75.47°, as displayed in Figure 3b [43]. However, these characteristic peaks do not appear in the XRD patterns of the P-CPCS-30 and P-CPCS-40 samples after soaking, indicating that NaCl is completely removed from the composite sheets via soaking in water. This further demonstrates the establishment of internal and external connected water paths in P-CPCS-30 and P-CPCS-40. As shown in Figure 4, the FTIR characteristic peaks of the raw PP mainly include 2917 cm^−1^, 1456 cm^−1^, 1376 cm^−1^, 1157 cm^−1^, and 973 cm^−1^ [44], and the carbon flakes are characterized by a peak of 1650 cm^−1^ [45]. It is clear that CPCS, P-CPCS-30, and P-CPCS-40 have the same characteristic peaks, which demonstrates that the chemical composition and vibrational structure of PP have not changed during the fabrication process of the composite sheet. P-CPCS-30 and P-CPCS-40 This further indicates that the porous structure is mainly constructed through the adhesion and mutual support of PP and carbon flakes.

**Figure 5:**
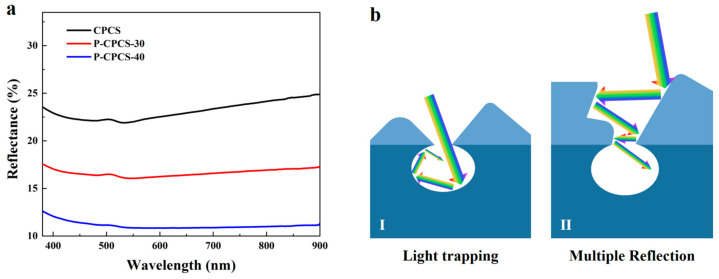
(**a**) Experimental reflectance spectra of CPCS, P-CPCS-30, and P-CPCS-40. (**b**) Schematic illustration showing light trapping by the porous structure of P-CPCS.

The reflectances of CPCS, P-CPCS-30, and P-CPCS-40 were measured in the visible-NIR wavelength range (380–900 nm) to study their optical properties, as shown in Figure 5a. Due to their porous structures, P-CPCS-30 and P-CPCS-40 show notably lower visible-NIR reflectance than CPCS. P-CPCS-40 has the lowest reflectance (close to 10%), which is attributed to its higher porosity compared to P-CPCS-30. These results indicate that light absorption is enhanced by greater porosity. Figure 5b schematically illustrates the light-trapping effect of this porous structure. On the one hand, the incident light that hits P-CPCS is trapped within the pores, leading to repeated reflections until complete light absorption is achieved. On the other hand, the rough and multi-dimensional structural surface of P-CPCS is also capable of inducing multiple reflections [46], which not only increases the optical path length but also improves the chance of light being trapped by the pores. Together, these factors mean that P-CPCS has excellent light absorption.

### 3.2. Photothermal Conversion by P-CPCS

**Figure 6:**
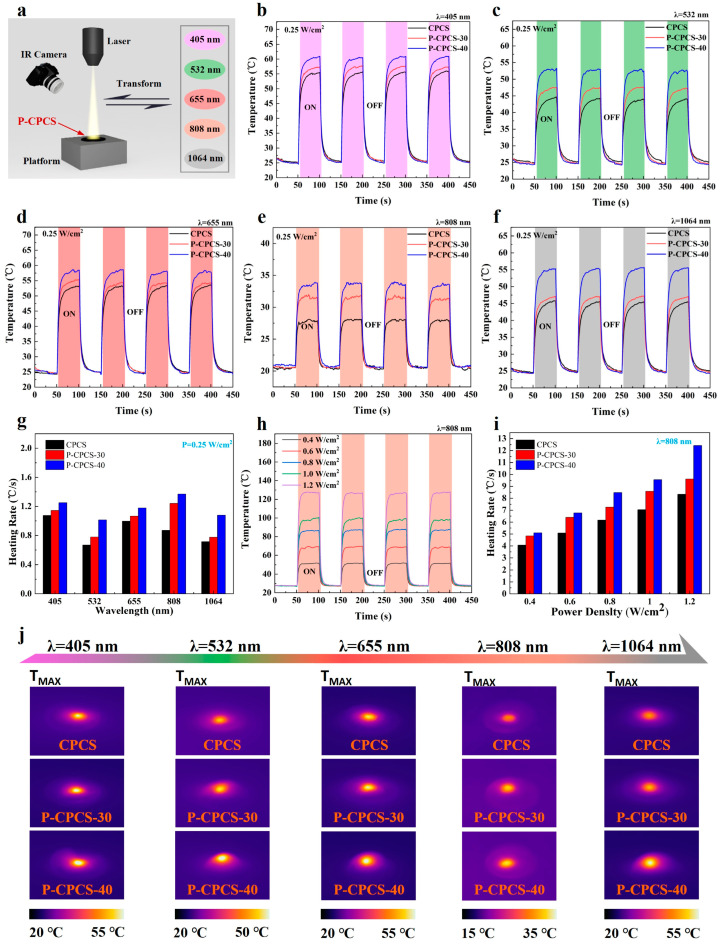
P-CPCS photothermal conversion evaluation. (**a**) Schematic diagram showing the photothermal conversion experiment. Temperature curves of samples under (**b**) 405 nm, (**c**) 532 nm, (**d**) 655 nm, (**e**) 808 nm, and (**f**) 1064 nm laser irradiation at a power density of 0.25 W/cm^2^. (**g**) Heating rates of the samples irradiated by 405, 532, 655, 808, and 1064 nm lasers at a power density of 0.25 W/cm^2^. (**h**) Temperature curves of P-CPCS-40 irradiated by 808 nm laser at power densities of 0.4, 0.6, 0.8, 1.0, and 1.2 W/cm^2^. (**i**) Heating rates of samples irradiated under 808 nm laser at different power densities. (**j**) IR images of CPCS, P-CPCS-30, and P-CPCS-40 irradiated by 405, 532, 655, 808, and 1064 nm lasers at a power density of 0.25 W/cm^2^.

P-CPCS has low reflectance in the visible-NIR range. Therefore, this material shows promise for photothermal conversion applications. In this study, CPCS, P-CPCS-30, and P-CPCS-40 were irradiated under the same conditions to evaluate their photothermal conversion properties. A schematic diagram of the setup used for this experiment is shown in Figure 6a. Each sample was irradiated by lasers with different wavelengths at the same intensity (0.25 W/cm^2^), and changes in temperature were determined using an IR camera.

The temperature curves of CPCS, P-CPCS-30, and P-CPCS-40 are displayed in Figure 6b–f, and the related IR images are provided in Figure 6j. Under irradiation at different wavelengths, the surface temperatures of all samples rapidly increase within 5 s due to light absorption by the carbon flakes. P-CPCS-40 exhibits increases in temperature ranging from 13.0 °C to 36.0 °C. CPCS shows the smallest increases in temperature, while those of P-CPCS-30 are between those of P-CPCS-40 and CPCS. Therefore, P-CPCS shows superb photothermal conversion performance for different laser wavelengths. Moreover, the enhanced performance of P-CPCS-40 compared to the other samples indicates that the porous structure helps improve the photothermal conversion properties. Moreover, higher porosity leads to a stronger photothermal effect. The heating rates of CPCS, P-CPCS-30, and P-CPCS-40 under laser irradiation are summarized in Figure 6g. Under each wavelength, P-CPCS-40 has a better heating rate than P-CPCS-30, while P-CPCS-30 has a better heating rate than CPCS. As the wavelength increases, the obtained heating rates do not show a clear pattern. Similarly, the temperature rise values displayed in Figure 6b–f also do not show a clear pattern as the wavelength increases, and the maximum temperature rise for P-CPCS is achieved at a wavelength of 405 nm. This may be related to the pore size distribution in P-CPCS and the photon energy of the laser, and the mechanism behind this phenomenon will be explored in further work. In any case, due to the added effect of the porous structure, significantly improved broadband photothermal conversion properties are achieved.

No loss of temperature is observed after cyclic irradiation, as displayed in Figure 6b–f. Thus, P-CPCS shows excellent photothermal conversion stability at low temperatures. To evaluate the high-temperature stability of P-CPCS-40, this sample was irradiated with different power densities using the 808 nm laser. As shown in Figure 6h, the rise in temperature is positively correlated with the laser power. The temperature of P-CPCS-40 increases to approximately 128 °C when irradiated at 1.2 W/cm^2^. More importantly, temperature loss is also not seen after cyclic irradiation at this temperature. This result indicates that P-CPCS also has excellent photothermal conversion stability at high temperatures. The heating rates of CPCS, P-CPCS-30, and P-CPCS-40 when irradiated with different power densities by the 808 nm laser are summarized in Figure 6i. As the laser power increases from 0.4 to 1.2 W/cm^2^, a nearly linear rise in heating rate is observed, and P-CPCS-40 achieves a high heating rate of 12.4 °C/s at 1.2 W/cm^2^. The heating rates of P-CPCS-30 and P-CPCS-40 are significantly higher than those of CPCS under different power densities. Combined with the results shown in Figure 6g, this indicates that the porous structure of P-CPCS significantly improves the sensitivity of photothermal conversion.

### 3.3. Evaporation Performance of P-CPCS

**Figure 7:**
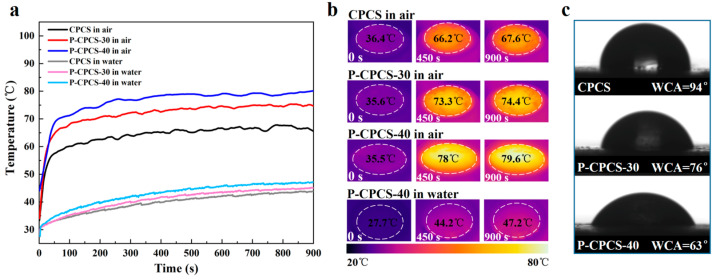
Evaluation of P-CPCS for solar steam generation. (**a**) Temperature curves of CPCS, P-CPCS-30, and P-CPCS-40 in water and air obtained under 1 sun irradiation. (**b**) IR images of CPCS, P-CPCS-30, and P-CPCS-40 in air and P-CPCS-40 in water under 1 sun irradiation from 0 to 900 s. (**c**) WCAs of CPCS, P-CPCS-30, and P-CPCS-40.

Due to its internal and external connected water paths and excellent photothermal conversion performance, P-CPCS offers excellent potential for application in solar steam generation. Therefore, to evaluate the photothermal conversion properties of P-CPCS under solar irradiation, the temperature changes of CPCS, P-CPCS-30, and P-CPCS-40 under 1 kW·m^−2^ solar irradiation were determined in water and air. Photothermal conversion performance in water was evaluated by completely immersing each sample under the water near the water surface. Figure 7a shows that P-CPCS exhibits a notably higher change in temperature than CPCS in both air and water. The IR images shown in Figure 7b provide a more intuitive view of the temperature changes obtained over a 900 s period under 1 kW·m^−2^ solar irradiation. P-CPCS-40 in air experiences a rise in temperature to 79.6 °C, while P-CPCS-40 in water shows a rise in temperature to 47.2 °C. The obtained temperature rises of 44.1 and 19.5 °C indicate that P-CPCS has outstanding photothermal conversion performance under solar irradiation. As shown in Figure 7c, the WCA of CPCS is 94°, while the WCAs of P-CPCS-30 and P-CPCS-40 are 76° and 63°, respectively. This indicates that the porous structure of P-CPCS improves its hydrophilicity, which facilitates the infiltration of the water around P-CPCS into its interior and upper surface. This water is then heated and evaporated under irradiation.

**Figure 8:**
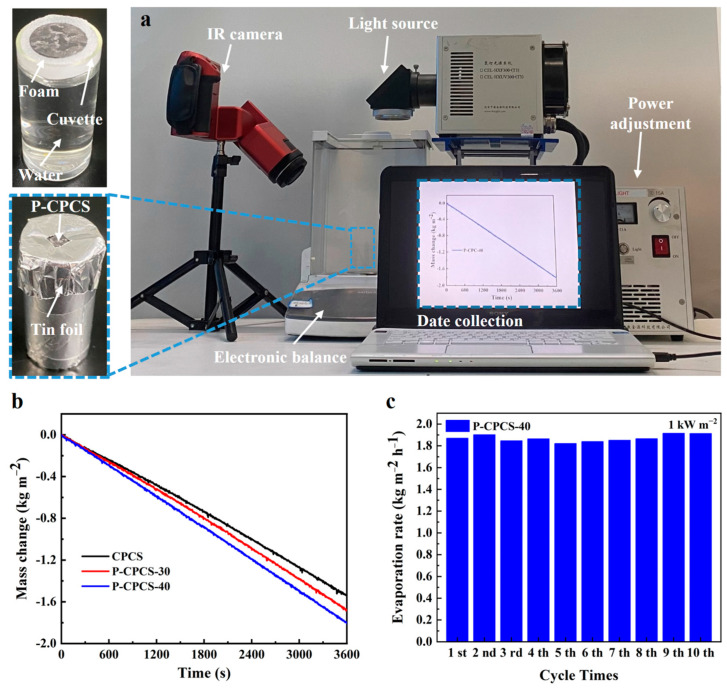
Evaporation performance of P-CPCS. (**a**) Physical image of the experimental setup for the solar-driven water evaporation experiment. (**b**) Changes in water mass achieved by CPCS, P-CPCS-30, and P-CPCS-40 under 1 sun irradiation. (**c**) Evaporation rates of P-CPCS-40 under 1 sun irradiation for 10 cycles.

The solar evaporation performance of P-CPCS was experimentally investigated to evaluate its potential for use in a solar steam generator. Figure 8a shows the physical image of the experimental setup. A foam block with a shallow circular groove was used to fix the sample at the center of the water surface to prevent the sample from drifting and, more importantly, to absorb the water in the cuvette to ensure the presence of water around the sample. Reflective tin foil was used to wrap the cuvette and cover the foam. Thus, only the sample surface was exposed to solar illumination, preventing the water in the foam block from being evaporated under solar irradiation. The cuvette was set on a high-precision electronic balance, and the change in mass was recorded on a laptop. The changes in the masses of CPCS, P-CPCS-30, and P-CPCS-40 under 1 kW m^−2^ solar irradiation are displayed in Figure 8b. CPCS clearly has the lowest evaporation rate, and P-CPCS-40 achieves the highest evaporation rate of 1.81 kg m^−2^ h^−1^. According to Equations (1)–(3) [47,48], the evaporation efficiency of P-CPCS-40 is 98.2%, and the calculations are provided in Note S1.

(1) $$\eta_{sv}=\frac{\dot{m}\times (H_{LV}+Q)}{E_{in}}$$

(2) $$H_{LV}=1.91846\times 10^{6}\times {\left[T_{fin}/(T_{fin}-33.91)\right]}^{2}$$

(3) $$Q=c\times (T_{fin}-T_{in})$$

where $\eta_{sv}$ represents the evaporation efficiency, $\dot{m}$ is defined as the evaporation rate under irradiation minus the evaporation rate in the dark (Figure S3), $H_{LV}$ is the latent heat required to vaporize water, Q is the heat required to raise the water temperature, $E_{in}$ is the incident light energy input, $T_{in}$ is the initial sample surface temperature, $T_{fin}$ is the average sample surface temperature during evaporation, and *c* represents the specific heat capacity of water. The high evaporation rate and efficiency of P-CPCS-40 are attributed to its high photothermal conversion performance and hydrophilicity.

**Figure 9:**
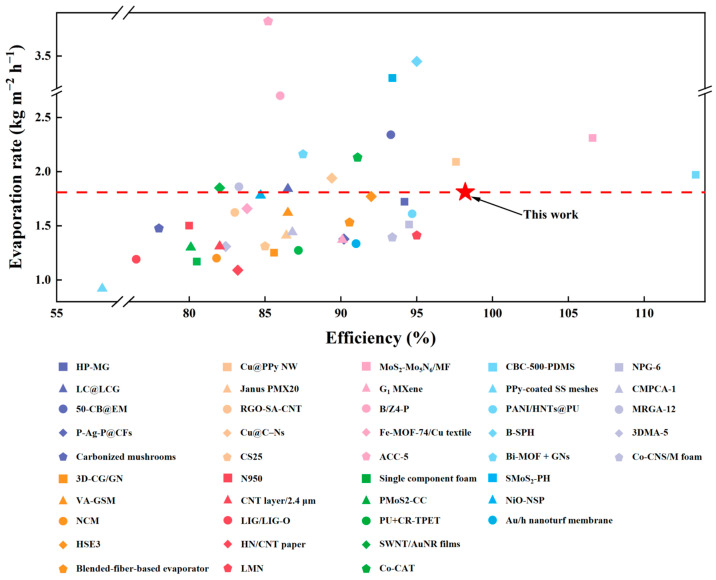
Comparison of evaporation rates and evaporation efficiencies reported for other absorber materials in previous studies.

The evaporation rate of P-CPCS-40 under 1 sun irradiation was measured for 10 cycles to evaluate its reusability. Figure 8c summarizes the evaporation rate per irradiation cycle (the change in the mass of water in each cycle is displayed in Figure S4). The evaporation rate does not significantly change across 10 cycles, demonstrating the good reusability of P-CPCS. This is ascribed to the stability of the self-supporting porous structure of P-CPCS and the good bonding of the carbon flakes to the PP. For comparison, the water evaporation performance of other reported absorbers such as porous materials, nanomaterials, and composite films under 1 sun irradiation is summarized in Figure 9 and Table S1. Among these absorbers, P-CPCS-40 is within the top 30% in terms of evaporation rate. Moreover, the evaporation efficiency of P-CPCS-40 is better than all listed materials other than MoS_2_-Mo_5_N_6_/MF and CBC-500-PDMS, demonstrating that P-CPCS-40 leads the way in achieving good evaporation performance. Although P-CPCS-40 does not exhibit the highest evaporation rate or efficiency amongst these absorber materials, P-CPCS has a low-cost, green, and non-polluting manufacturing process. Moreover, the evaporation performance could still potentially be improved by optimizing the porous structure or increasing the carbon flake content. Overall, P-CPCS has important research significance and practical application value.

### 3.4. Desalination and Sewage Treatment

**Figure 10:**
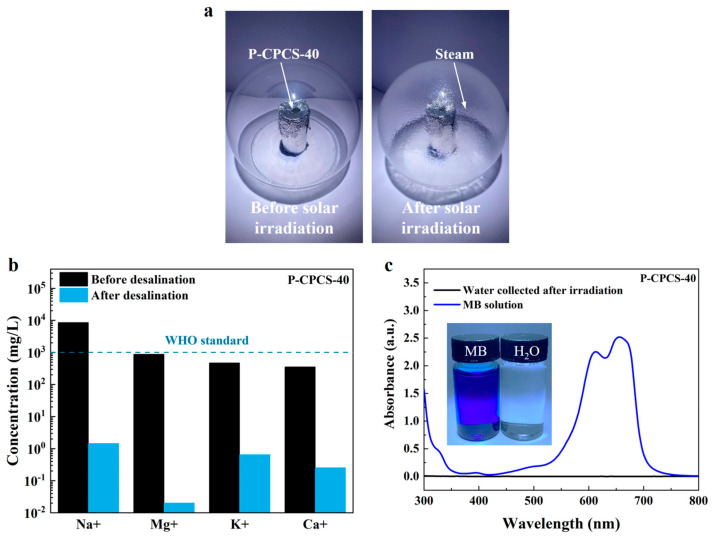
Desalination and sewage treatment performance of P-CPCS. (**a**) Schematic illustration of experimental setup utilized for evaporation and steam collection. (**b**) Na^+^, Mg^2+^, K^+^, and Ca^2+^ concentrations in simulated seawater before irradiation and in the water collected during evaporation by P-CPCS-40 (WHO standard: dashed line). (**c**) UV–Vis–NIR absorbance spectra of MB solution before irradiation and the water collected during evaporation by P-CPCS-40.

The desalination performance of P-CPCS when utilized for solar steam generation in seawater was investigated by performing evaporation experiments using simulated seawater instead of pure water and collecting the water after steam condensation. As shown in Figure 10a, the steam generation source (simulated seawater with P-CPCS-40 on the surface) was covered with glass under 1 sun irradiation. As a result, the steam evaporating from the P-CPCS-40 surface condensed on the inner glass surface. The concentrations of Na^+^, Mg^2+^, K^+^, and Ca^2+^ in the simulated seawater were measured before irradiation and in the water collected during the evaporation process, as indicated in Figure 10b. The ion concentrations of the water evaporated from the P-CPCS-40 surface are nearly three orders of magnitude lower than those of the simulated seawater. Moreover, these ion concentrations are significantly lower than the World Health Organization (WHO) drinking water standard. These results show the superb desalination performance of -CPCS.

The practical application of P-CPCS was further explored in a sewage treatment application. A methylene blue (MB) solution was used as the simulated sewage, and an evaporation experiment was performed using the same procedure as that described for seawater evaporation. The UV–Vis–NIR absorbance spectra of the MB solution before irradiation and the water collected after irradiation are displayed in Figure 10c. Two strong absorption peaks are observed at 610 nm and 650 nm in the MB solution, but these absorption peaks almost completely disappear in the water collected after irradiation. This demonstrates the excellent sewage treatment performance of P-CPCS.

## 4. Conclusions

In this study, a low-cost and green method that combines ultrasonic pressing and salt dissolution for the fabrication of porous composite structures is presented. This method was utilized to successfully prepare porous carbon polypropylene composite sheets (P-CPCS). The relevant results are as follows:

(1) The P-CPCS samples have sufficient strength when prepared with NaCl volume ratios as high as 40%. P-CPCS has a well-distributed porous structure with internal and external connected water paths. The CPCS, P-CPCS-30, and P-CPCS-40 mainly show differences in porosity within the pore size range of 10 nm to 10 μm. Differences in light reflectance properties are primarily attributed to this difference in porosity. In the visible-NIR wavelength region, P-CPCS-40 has a low reflectance of nearly 10%.
(2) P-CPCS has an enhanced porous structure, leading to significantly better photothermal properties compared to CPCS. The P-CPCS-40 sample rapidly reaches a temperature of 128 °C at a heating rate of 12.4 °C/s when irradiated at 1.2 W/cm^2^ with an 808 nm laser.
(3) P-CPCS-40 achieves an evaporation rate as high as 1.81 kg m^−2^ h^−1^ and evaporation efficiency of 98.2% under 1 sun irradiation. Compared to previously reported absorbers, P-CPCS-40 ranks among the top in terms of evaporation efficiency.
(4) Desalination and sewage treatment experiments were performed, confirming the potential of P-CPCS for practical applications. We believe that the application of P-CPCS is not only limited to these fields. Due to its promising performance, P-CPCS could potentially be applied in fields such as rehabilitative physiotherapy, photocatalysis, and photothermal imaging.

## Data Availability

The original contributions presented in the study are included in the article/Supplementary Materials, further inquiries can be directed to the corresponding author/s.

## Supplementary Materials

The following supporting information can be downloaded at: https://www.mdpi.com/article/10.3390/polym16192813/s1, Note S1: Calculation of solar-vapor conversion efficiency; Figure S1: SEM images of carbon flakes; Figure S2: (a) SEM image of NaCl powder. (b) Particle size analysis image of NaCl powder; Figure S3: Mass change of water for P-CPCS-40 in the dark condition; Figure S4: Mass changes of water for P-CPCS-40 evaporator under 1 kW m^−2^ solar irradiation from 1 to 10 cycles; Table S1: Water evaporation performance of other reported absorbers in previous studies.
