# Supplementary material for: Fabrication of Low-Cost Porous Carbon Polypropylene Composite Sheets with High Photothermal Conversion Performance for Solar Steam Generation
Source: Polymers (Basel). 2024 Oct 4;16(19):2813. doi: 10.3390/polym16192813 (PMC11479202; doi:10.3390/polym16192813)
Supplement: Supplementary file 1 [file polymers-16-02813-s001.zip › polymers-3232274-supplementary.pdf]

Supplementary Material

Fabrication of low-cost porous carbon polypropylene composite sheets with high photothermal conversion performance for solar steam generation

Shuqing Xu[1], Shiyun Wu [2*], Bin Xu[1], Jiang Ma[1], Jianjun Du[2], Jianguo Lei[1]

[1] *College of Mechatronics and Control Engineering, Shenzhen University, Shenzhen 518061, China*

[2] *School of Mechanical Engineering and Automation, Harbin Institute of Technology Shenzhen, Shenzhen, 518055, China*

[*] **Corresponding author E-mail address**: wusy626@163.com (Shiyun Wu)

Note S1. Calculation of solar-vapor conversion efficiency.

The solar-vapor conversion efficiency can be calculated by the following equations [1].

$$\eta_{SV} = \dot{m}(H_{LV} + Q)/E_{in} \tag{1}$$

$$\dot{m} = m_{actual} - m_{dark} \tag{2}$$

$$H_{LV} = 1.91846 * 10^6 * [T_{fin}/(T_{fin} - 33.91)]^2 \tag{3}$$

$$Q = c * (T_{fin} - T_{in}) \tag{4}$$

where η_{sv} represents the evaporation efficiency, $\dot{m}$ is defined as the evaporation rate under irradiation minus the evaporation rate in the dark (Fig. S3), H_{LV} is the latent heat required to vaporize water (J/kg), Q is the heat required to raise the water temperature, E_{in} is the incident light energy input (kJ/m2h), m_{actual} is the evaporation rate under irradiation, m_{dark} is the evaporation rate under dark condition, T_{in} is the initial sample surface temperature, T_{fin} is the average sample surface temperature during evaporation, and c represents the specific heat capacity of water (4.2 J/gK).

For the P-CPCS-40 evaporator under 1 sun irradiation, m_{actual}= 1.81 kg m-2 h-1, m_{dark}= 0.3372 kg m-2 h-1 (Fig. S3), T_{fin}=320.35 K, T_{in}=300.85 K, and E_{in}= 360000 kJ/m2h. Based on the above equations, η_{SV} can be calculated to be 98.2%.

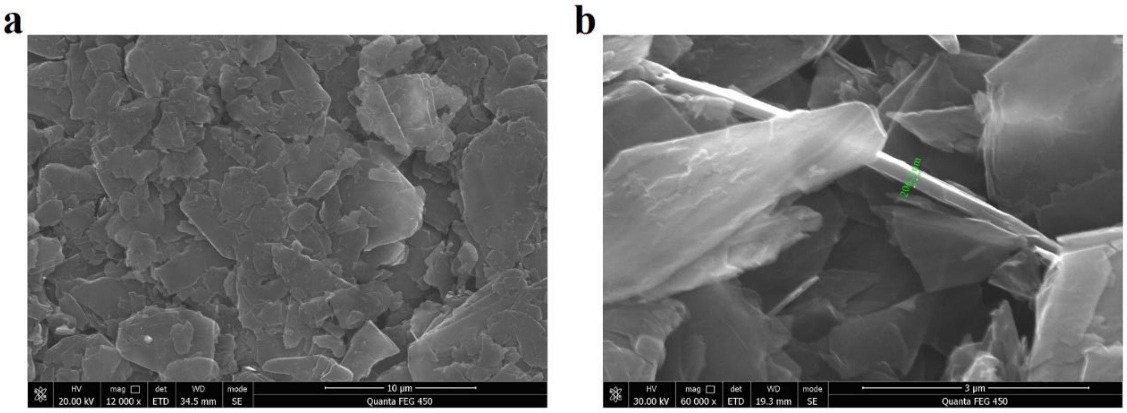

Figure S1. SEM images of carbon flakes.

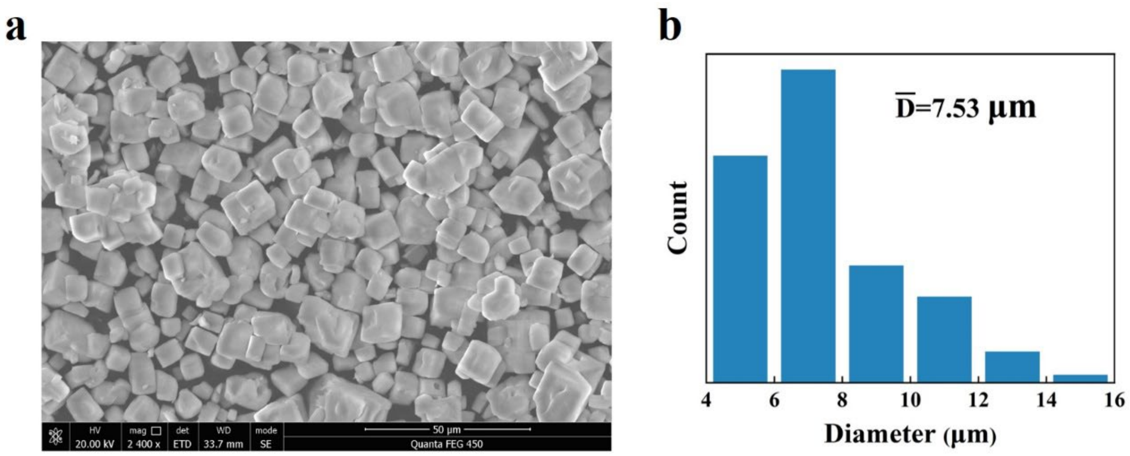

Figure S2. (a) SEM image of NaCl powder. (b) Particle size analysis image of NaCl powder.

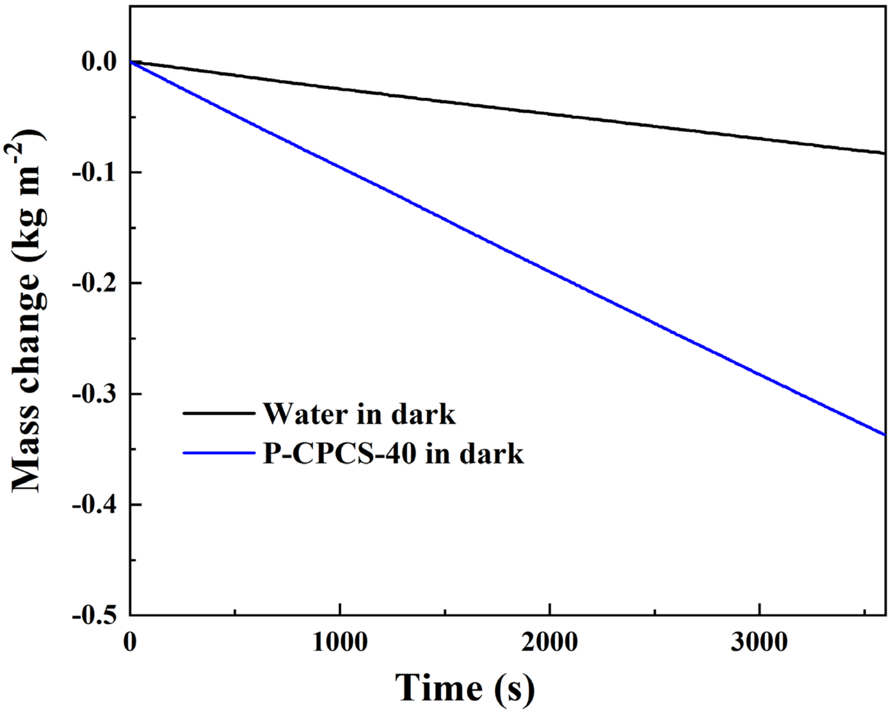

Figure S3. Mass change of water for P-CPCS-40 in the dark condition.

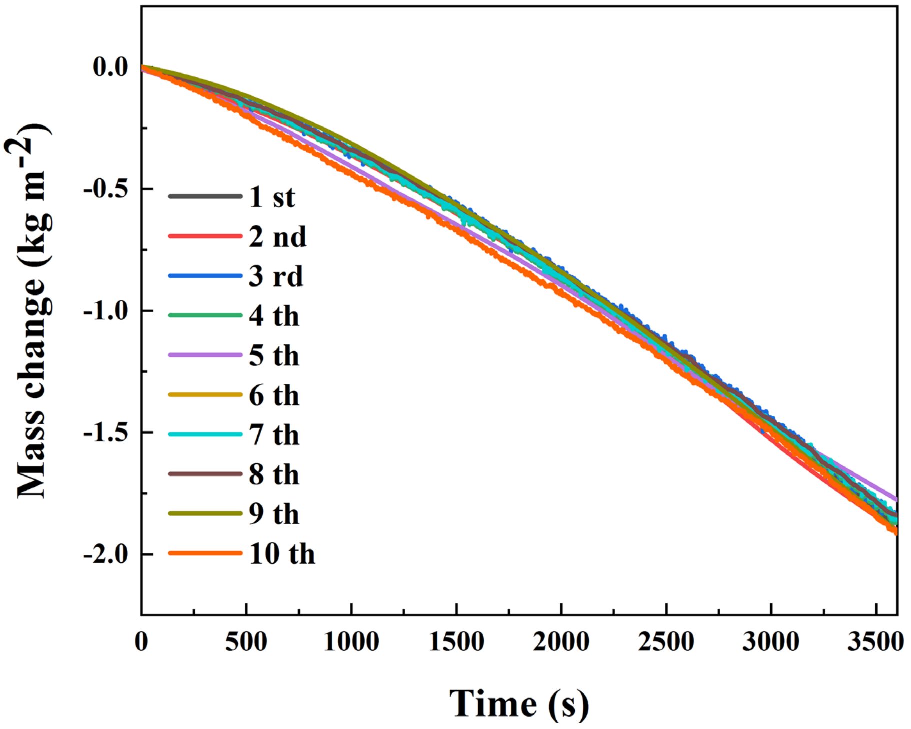

Figure S4. Mass changes of water for P-CPCS-40 evaporator under 1 kWm−2 solar irradiation from 1 to 10 cycles.

Table S1. Water evaporation performance of other reported absorbers in previous studies.

**Materials**	**T_{max} in air (℃)**	**Heating rate in air (℃/s)**	**T_{max} in water (℃)**	**Heating rate in water (℃/s)**	**Evaporation rate (kg $m^{-2}h^{-1}$)**	**Evaporation efficiency (%)**	**Reference**
P-CPC-40	79.6	0.1764	47.2	0.078	1.81	98.2	This work
HP-MG	41.3	0.0118	35.3	0.0143	1.72	94.2	[2]
LC@LCG	39	0.01			1.84	86.5	[3]
50-CB@EM	60	0.125	-	-	2.34	93.29	[4]
P-Ag-P@CFs	41.1	0.087	-	-	1.378	90.2	[5]
Carbonized mushrooms	38	0.0270	-	-	1.475	78	[6]
Cu@PPy NW	78	1.16	-	-	2.09	97.6	[7]
Janus PMX20	72.1	0.2589	46.5	0.1472	1.41±0.04	86.4	[8]
RGO-SA-CNT	-	-	40	0.0042	1.622	83	[9]
Cu@C–Ns	73.5	0.0539	-	-	1.94	89.4	[10]
CS25	-	-	47	0.225	1.31	85	[11]
MoS_2-Mo_5N_6/MF	103	0.6667	51	0.1556	2.31	106.6	[12]
G_1 MXene	62.8	0.1227	-	-	1.37	90.1	[13]
B/Z4-P	45.3	-	-	-	2.7	86	[14]
Fe-MOF-74/Cu textile	52.7	0.4776	40.4	0.1533	1.659	83.81	[15]

Materials	T_{max} in air (℃)	Heating rate in air (℃/s)	T_{max} in water (℃)	Heating rate in water (℃/s)	Evaporation rate (kg $m^{-2}h^{-1}$)	Evaporation efficiency (%)	Reference
ACC-5	32	0.0233	-	-	3.82	85.2	[16]
CBC-500-PDMS	76	0.46	-	-	1.97	113.4	[17]
PPy-coated SS meshes	55	3.3	39	0.0023	0.92	58	[18]
PANI/HNTs@PU	-	-	38	0.0111	1.61	94.7	[19]
B-SPH	-	-	30.3	0.0057	3.45	95	[20]
Bi-MOF + GNs	94.9	0.783			2.16	87.5	[21]
NPG-6	37	0.0394	-	-	1.51	94.5	[22]
CMPCA-1	37.5	0.0158	-	-	1.4406	86.8	[23]
MRGA-12	75.9	0.2733	-	-	1.86	83.28	[24]
3DMA-5	80	0.31	39	0.025	1.309	82.4	[25]
Co-CNS/M foam	69.8	0.73	33.2	0.0567	1.393	93.39	[26]
3D-CG/GN	36.5	0.055	-	-	1.25	85.6	[27]
VA-GSM	-	-	-	-	1.62	86.5	[28]
NCM	59	0.1	-	-	1.2	81.8	[29]
HSE3	-	-	45.5	0.0057	1.77	92	[30]
Blended-fiber-based evaporator	44.5	0.0694	-	-	1.53	90.57	[31]
N950	100	0.2533	40	0.0533	1.5	80	[32]
CNT layer/2.4 μm	72	0.1733	-	-	1.31	82	[33]
LIG/LIG-O	-	-	29.4	0.0056	1.191	76.5	[34]
HN/CNT paper	80	0.4167	37	0.02	1.09	83.2	[35]
LMN	66.2	0.0117	-	-	1.41	95	[36]
Single component foam	46.7	0.0063	30.6	0.0018	1.1687	80.5	[37]
$PMoS_2$-CC	-	-	43.2	0.0513	1.3	80.1	[38]
PU+CR-TPET	77	0.0867	43	0.0056	1.272	87.2	[39]
SWNT/AuNR films	-	-	-	-	1.85	82	[40]
Co-CAT	106.6	0.118	-	-	2.13	91.1	[41]
$SMoS_2$-PH	-	-	37.7	0.0074	3.297	93.4	[42]
NiO-NSP	37.7	0.0275	-	-	1.78	84.7	[43]
Au/h nanoturf membrane	48.4	0.61	-	-	1.334±0.045	91	[44]
SWNT–MoS_2 film	-	-	-	-	-	81	[45]
FTCS-PDA/BNC	78	5.4	33	0.015	-	-	[46]
SG3	-	-	42.6	0.004	2.21	-	[47]
SSA	91	0.1725	-	-	-	-	[48]
R-NFAs	65	0.75	40.2	0.0169	1.31	-	[49]
NVGQF	80.7	0.3713	-	-	-	-	[50]
TPAD-COF-BF_2	58	0.165	41.5	0.0103	1.19	-	[51]

Note: All data in the table were obtained under 1 sun irradiation.

References

[1] Md.N.A.S. Ivan, A.M. Saleque, S. Ahmed, P.K. Cheng, J. Qiao, T.I. Alam, Y.H. Tsang, Waste Egg Tray and Toner-Derived Highly Efficient 3D Solar Evaporator for Freshwater Generation, ACS Appl. Mater. Interfaces 14 (2022) 7936–7948. https://doi.org/10/gtppks.

[2] J. Fu, Z. Li, X. Li, F. Sun, L. Li, H. Li, J. Zhao, J. Ma, Hierarchical porous metallic glass with strong broadband absorption and photothermal conversion performance for solar steam generation, Nano Energy 106 (2023) 108019. https://doi.org/10/grsrwb.

[3] C. Lei, J. Park, W. Guan, Y. Zhao, K.P. Johnston, G. Yu, Biomimetically Assembled Sponge‐Like Hydrogels for Efficient Solar Water Purification, Adv. Funct. Mater. 33 (2023) 2303883. https://doi.org/10/gr853d.

[4] W. Wei, C. Li, X. Qi, L. He, X. Zhang, J. He, Z. Gao, Solid waste reutilization of FCC petroleum coke for solar evaporation as a photothermal conversion material, Colloids Surf., A 692 (2024) 133968. https://doi.org/10/gtzw5p.

[5] Y. Xu, B. Tang, X. Fang, T. Ma, D. Yu, G. Zhou, Z. Zhang, A Facile Approach to Fabricate Sustainable and Large-Scale Photothermal Polydopamine-Coated Cotton Fabrics for Efficient Interfacial Solar Steam Generation, Ind. Eng. Chem. Res. 61 (2022) 18109–18120. https://doi.org/10.1021/acs.iecr.2c03477.

[6] N. Xu, X. Hu, W. Xu, X. Li, L. Zhou, S. Zhu, J. Zhu, Mushrooms as Efficient Solar Steam-Generation Devices, Adv. Mater. 29 (2017) 1606762. https://doi.org/10.1002/adma.201606762.

[7] W. Wang, X. Yan, J. Geng, N. Zhao, L. Liu, T. Vogel, Q. Guo, L. Ge, B. Luo, Y. Zhao, Engineering a Copper@Polypyrrole Nanowire Network in the Near Field for Plasmon-Enhanced Solar Evaporation, ACS Nano 15 (2021) 16376–16394. https://doi.org/10.1021/acsnano.1c05789.

[8] B. Zhang, Q. Gu, C. Wang, Q. Gao, J. Guo, P.W. Wong, C.T. Liu, A.K. An, Self-Assembled Hydrophobic/Hydrophilic Porphyrin-$Ti_3C_2T_x$ MXene Janus Membrane for Dual-Functional Enabled Photothermal Desalination, ACS. Appl. Mater. Interfaces 13 (2021) 3762–3770. https://doi.org/10.1021/acsami.0c16054.

[9] X. Hu, W. Xu, L. Zhou, Y. Tan, Y. Wang, S. Zhu, J. Zhu, Tailoring Graphene Oxide-Based Aerogels for Efficient Solar Steam Generation under One Sun, Adv. Mater. 29(2017) 1604031. https://doi.org/10.1002/adma.201604031.

[10] X. Wu, C. Li, Z. Zhang, Y. Cao, J. Wang, X. Tian, Z. Liu, Y. Shen, M. Zhang, W. Huang, Nitrogen‐doped microporous graphite‐enhanced copper plasmonic effect for solar evaporation, Carbon Energy 6 (2024) e466. https://doi.org/10.1002/cey2.466.

[11] L. Zhu, M. Gao, C.K.N. Peh, X. Wang, G.W. Ho, Self‐Contained Monolithic Carbon Sponges for Solar‐Driven Interfacial Water Evaporation Distillation and Electricity Generation, Adv. Energy Mater. 8 (2018) 1702149. https://doi.org/10/gdtd5m.

[12] L. Wang, D. Liu, L. Jiang, Y. Ma, G. Yang, Y. Qian, W. Lei, Advanced 2D–2D heterostructures of transition metal dichalcogenides and nitrogen-rich nitrides for solar water generation, Nano Energy 98 (2022) 107192. https://doi.org/10/gtzw5m.

[13] K. Li, T. Chang, Z. Li, H. Yang, F. Fu, T. Li, J.S. Ho, P. Chen, Biomimetic MXene Textures with Enhanced Light‐to‐Heat Conversion for Solar Steam Generation and Wearable Thermal Management, Adv. Energy Mater. 9 (2019) 1901687.

https://doi.org/10.1002/aenm.201901687.

[14] Y. Lu, D. Fan, Z. Shen, H. Zhang, H. Xu, X. Yang, Design and performance boost of a MOF-functionalized-wood solar evaporator through tuning the hydrogen-bonding interactions, Nano Energy 95 (2022) 107016. https://doi.org/10/gtzw5g.

[15] J. Wang, W. Wang, J. Li, X. Mu, X. Yan, Z. Wang, J. Su, T. Lei, C. Wang, Universal Strategy to Prepare a Flexible Photothermal Absorber Based on Hierarchical Fe-MOF-74 toward Highly Efficient Solar Interfacial Seawater Desalination, ACS. Appl. Mater. Interfaces 13 (2021) 45944–45956. https://doi.org/10.1021/acsami.1c11176.

[16] D.V. Lam, U.N.T. Nguyen, D.T. Dung, C. Kim, M. Lim, J.-H. Kim, S.-M. Lee, Shape-transformable long-lasting superhydrophilic carbon cloth for sustainable solar vapor generation, Chem. Eng. J. 481 (2024) 148475. https://doi.org/10/gtzw5h.

[17] G. Chen, Z. Jiang, A. Li, X. Chen, Z. Ma, H. Song, Cu-based MOF-derived porous carbon with highly efficient photothermal conversion performance for solar steam evaporation, J. Mater. Chem. A. 9 (2021) 16805–16813. https://doi.org/10/gtppt3.

[18] L. Zhang, B. Tang, J. Wu, R. Li, P. Wang, Hydrophobic Light-to-Heat Conversion Membranes with Self-Healing Ability for Interfacial Solar Heating, Adv. Mater. 27 (2015) 4889–4894. https://doi.org/10/f3ctcf.

[19] J. Han, W. Xing, J. Yan, J. Wen, Y. Liu, Y. Wang, Z. Wu, L. Tang, J. Gao, Stretchable and Superhydrophilic Polyaniline/Halloysite Decorated Nanofiber Composite Evaporator for High Efficiency Seawater Desalination, Adv. Fiber Mater. 4 (2022) 1233–1245. https://doi.org/10/gtzw5j.

[20] C. Lei, J. Park, W. Guan, Y. Zhao, K.P. Johnston, G. Yu, Biomimetically Assembled Sponge ‐ Like Hydrogels for Efficient Solar Water Purification, Adv. Funct. Mater. 33 (2023) 2303883. https://doi.org/10.1002/adfm.202303883.

[21] Z. Fan, J. Liu, H. Liu, L. Liu, Y. She, X. Wen, H. Wang, G. Hu, R. Niu, J. Gong, Synergism of solar-driven interfacial evaporation and photo-Fenton Cr(VI) reduction by sustainable Bi-MOF-based evaporator from waste polyester, J. Energy Chem. 94 (2024) 527–540. https://doi.org/10/gtzw5n.

[22] Y. Zhang, Y. Wang, B. Yu, K. Yin, Z. Zhang, Hierarchically Structured Black Gold Film with Ultrahigh Porosity for Solar Steam Generation, Adv. Mater. 34 (2022) 2200108. https://doi.org/10.1002/adma.202200108.

[23] P. Mu, Z. Zhang, W. Bai, J. He, H. Sun, Z. Zhu, W. Liang, A. Li, Superwetting Monolithic Hollow ‐ Carbon ‐ Nanotubes Aerogels with Hierarchically Nanoporous Structure for Efficient Solar Steam Generation, Adv. Energy Mater. 9 (2019) 1802158. https://doi.org/10/gtpptc.

[24] G. Zhang, Y. Zhang, J. Jiang, Robust and multifunctional MXene/rGO composite aerogels toward highly efficient solar-driven interfacial evaporation and wastewater treatment, Sep. Purif. Technol. 347 (2024) 127588. https://doi.org/10/gtzw5s.

[25] X. Zhao, X.-J. Zha, J.-H. Pu, L. Bai, R.-Y. Bao, Z.-Y. Liu, M.-B. Yang, W. Yang, Macroporous three-dimensional MXene architectures for highly efficient solar steam generation, J. Mater. Chem. A 7 (2019) 10446–10455. https://doi.org/10.1039/c9ta00176j.

[26] X. Fan, Y. Yang, X. Shi, Y. Liu, H. Li, J. Liang, Y. Chen, A MXene ‐ Based Hierarchical Design Enabling Highly Efficient and Stable Solar ‐ Water Desalination with Good Salt Resistance, Adv. Funct. Mater. 30 (2020) 2007110. https://doi.org/10.1002/adfm.202007110.

[27] Y. Li, T. Gao, Z. Yang, C. Chen, W. Luo, J. Song, E. Hitz, C. Jia, Y. Zhou, B. Liu, B. Yang, L. Hu, 3D‐Printed, All‐in‐One Evaporator for High‐Efficiency Solar Steam Generation under 1 Sun Illumination, Adv. Mater. 29 (2017) 1700981. https://doi.org/10.1002/adma.201700981.

[28] P. Zhang, J. Li, L. Lv, Y. Zhao, L. Qu, Vertically Aligned Graphene Sheets Membrane for Highly Efficient Solar Thermal Generation of Clean Water, ACS Nano 11 (2017) 5087–5093. https://doi.org/10.1021/acsnano.7b01965.

[29] Y. Yuan, C. Dong, J. Gu, Q. Liu, J. Xu, C. Zhou, G. Song, W. Chen, L. Yao, D. Zhang, A Scalable Nickel–Cellulose Hybrid Metamaterial with Broadband Light Absorption for Efficient Solar Distillation, Adv. Mater. 32 (2020) 1907975. https://doi.org/10.1002/adma.201907975.

[30] W. Lei, S. Khan, L. Chen, N. Suzuki, C. Terashima, K. Liu, A. Fujishima, M. Liu, Hierarchical structures hydrogel evaporator and superhydrophilic water collect device for efficient solar steam evaporation, Nano Res. 14 (2021) 1135–1140. https://doi.org/10/gtpptg.

[31] Q. Liu, X. Liu, G. Chen, P. Feng, Y. Xiong, M. An, C. Shao, X. Zhu, R. Wang, J. Sun, J. Sun, C. Guo, S. Bi, S. Li, A robust and low-cost blended-fiber-based evaporator with high efficiency for solar desalination, Desalination 583 (2024) 117715. https://doi.org/10/gtzw5q.

[32] Y. Ito, Y. Tanabe, J. Han, T. Fujita, K. Tanigaki, M. Chen, Multifunctional Porous Graphene for High-Efficiency Steam Generation by Heat Localization, Adv. Mater. 27 (2015) 4302–4307. https://doi.org/10/f278nk.

[33] Y. Wang, L. Zhang, P. Wang, Self-Floating Carbon Nanotube Membrane on Macroporous Silica Substrate for Highly Efficient Solar-Driven Interfacial Water Evaporation, ACS Sustainable Chem. Eng. 4 (2016) 1223–1230. https://doi.org/10/f8c5vc.

[34] D.-D. Han, Z.-D. Chen, J.-C. Li, J.-W. Mao, Z.-Z. Jiao, W. Wang, W. Zhang, Y.-L. Zhang, H.-B. Sun, Airflow Enhanced Solar Evaporation Based on Janus Graphene Membranes with Stable Interfacial Floatability, ACS Appl. Mater. Interfaces 12 (2020) 25435–25443. https://doi.org/10.1021/acsami.0c05401.

[35] Z. Xiong, Y. Zhu, D. Qin, F. Chen, R. Yang, Flexible Fire‐Resistant Photothermal Paper Comprising Ultralong Hydroxyapatite Nanowires and Carbon Nanotubes for Solar Energy‐Driven Water Purification, Small 14 (2018) 1803387. https://doi.org/10/gtpptb.

[36] Y. Lu, H. Zhang, Y. Wang, X. Zhu, W. Xiao, H. Xu, G. Li, Y. Li, D. Fan, H. Zeng, Z. Chen, X. Yang, Solar‐Driven Interfacial Evaporation Accelerated Electrocatalytic Water Splitting on 2D Perovskite Oxide/MXene Heterostructure, Adv. Funct. Mater. 33 (2023) 2215061. https://doi.org/10/gr85zn.

[37] Q. Chen, Z. Pei, Y. Xu, Z. Li, Y. Yang, Y. Wei, Y. Ji, A durable monolithic polymer foam for efficient solar steam generation, Chem. Sci. 9 (2018) 623–628. https://doi.org/10.1039/c7sc02967e.

[38] Z. Guo, G. Wang, X. Ming, T. Mei, J. Wang, J. Li, J. Qian, X. Wang, PEGylated Self-Growth MoS_2 on a Cotton Cloth Substrate for High-Efficiency Solar Energy Utilization, ACS Appl. Mater. Interfaces 10 (2018) 24583–24589. https://doi.org/10/gd2qf5.

[39] G. Chen, J. Sun, Q. Peng, Q. Sun, G. Wang, Y. Cai, X. Gu, Z. Shuai, B.Z. Tang, Biradical‐Featured Stable Organic‐Small‐Molecule Photothermal Materials for Highly Efficient Solar‐Driven Water Evaporation, Adv. Mater. 32 (2020) 1908537. https://doi.org/10.1002/adma.201908537.

[40] Y. Yang, X. Yang, L. Fu, M. Zou, A. Cao, Y. Du, Q. Yuan, C.-H. Yan, Two-Dimensional Flexible Bilayer Janus Membrane for Advanced Photothermal Water Desalination, ACS Energy Lett. 3 (2018) 1165–1171. https://doi.org/10.1021/acsenergylett.8b00433.

[41] P. He, H. Lan, H. Bai, Y. Zhu, Z. Fan, J. Liu, L. Liu, R. Niu, Z. Dong, J. Gong, Rational construction of "all-in-one" metal-organic framework for integrated solar steam generation and advanced oxidation process, Appl. Catal., B 337 (2023) 123001. https://doi.org/10/gtzw5k.

[42] P. Liu, Y. Hu, X. Li, L. Xu, C. Chen, B. Yuan, M. Fu, Enhanced Solar Evaporation Using a Scalable MoS_2 - Based Hydrogel for Highly Efficient Solar Desalination, Angew. Chem., Int. Ed. 61 (2022) e202208587. https://doi.org/10.1002/anie.202208587.

[43] R. Song, N. Zhang, P. Wang, H. Ding, S. Li, Three-dimensional NiO/Ni self-floating porous composite materials for efficient solar interfacial evaporation, Colloids Surf., A 695 (2024) 134261. https://doi.org/10.1016/j.colsurfa.2024.134261.

[44] J.U. Kim, S.J. Kang, S. Lee, J. Ok, Y. Kim, S.H. Roh, H. Hong, J.K. Kim, H. Chae, S.J. Kwon, T. Kim, Omnidirectional, Broadband Light Absorption in a Hierarchical Nanoturf Membrane for an Advanced Solar - Vapor Generator, Adv. Funct. Mater. 30 (2020) 2003862. https://doi.org/10/gjwbk9.

[45] X. Yang, Y. Yang, L. Fu, M. Zou, Z. Li, A. Cao, Q. Yuan, An Ultrathin Flexible 2D Membrane Based on Single - Walled Nanotube–MoS_2 Hybrid Film for High - Performance Solar Steam Generation, Adv. Funct. Mater. 28 (2018) 1704505. https://doi.org/10.1002/adfm.201704505.

[46] X. Wu, S. Cao, D. Ghim, Q. Jiang, S. Singamaneni, Y.-S. Jun, A thermally engineered polydopamine and bacterial nanocellulose bilayer membrane for photothermal membrane distillation with bactericidal capability, Nano Energy 79 (2021) 105353. https://doi.org/10/grmx93.

[47] F. Li, N. Li, S. Wang, L. Qiao, L. Yu, P. Murto, X. Xu, Self - Repairing and Damage - Tolerant Hydrogels for Efficient Solar - Powered Water Purification and Desalination, Adv. Funct. Mater. 31 (2021) 2104464. https://doi.org/10/gtpptd.

[48] Y. Li, C. Lin, Z. Wu, Z. Chen, C. Chi, F. Cao, D. Mei, H. Yan, C.Y. Tso, C.Y.H. Chao, B. Huang, Solution - Processed All - Ceramic Plasmonic Metamaterials for Efficient Solar–Thermal Conversion over 100–727°C, Adv. Mater. 33 (2021) 2005074. https://doi.org/10.1002/adma.202005074.

[49] X. Dong, Y. Si, C. Chen, B. Ding, H. Deng, Reed Leaves Inspired Silica Nanofibrous Aerogels with Parallel-Arranged Vessels for Salt-Resistant Solar Desalination, ACS Nano 15 (2021) 12256–12266. https://doi.org/10.1021/acsnano.1c04035.

[50] Y. Cheng, S. Cheng, B. Chen, J. Jiang, C. Tu, W. Li, Y. Yang, K. Huang, K. Wang, H. Yuan, J. Li, Y. Qi, Z. Liu, Graphene Infrared Radiation Management Targeting Photothermal Conversion for Electric-Energy-Free Crude Oil Collection, J. Am. Chem. Soc. 144 (2022) 15562–15568. https://doi.org/10.1021/jacs.2c04454.

[51] X. Yan, S. Lyu, X. Xu, W. Chen, P. Shang, Z. Yang, G. Zhang, W. Chen, Y. Wang, L. Chen, Superhydrophilic 2D Covalent Organic Frameworks as Broadband Absorbers for Efficient Solar Steam Generation, Angew. Chem., Int. Ed. 61 (2022) e202201900. https://doi.org/10/gpk9rk.
